# Psychosocial needs of women undergoing perinatal intimate examinations

**DOI:** 10.4102/hsag.v30i0.3179

**Published:** 2025-11-21

**Authors:** Ntsoaki M. Tshabalala, Mariatha Yazbek, Carin Maree

**Affiliations:** 1 Department of Nursing Science, Faculty of Health Sciences, University of Pretoria, Pretoria, South Africa; 2 Department of Nursing, Faculty of Health Sciences, Sefako Makgatho University, Pretoria, South Africa

**Keywords:** guidelines, holistic care, psychosocial needs, perinatal care, intimate examinations

## Abstract

**Background:**

Intimate examinations are a vital part of midwifery care during pregnancy, labour, and the postnatal period. Women’s experiences of these procedures vary greatly, and poor communication can lead to significant distress, particularly for first-time mothers. Despite its importance, psychological health during such examinations is often overlooked.

**Aim:**

The study aims to explore the psychosocial needs of women undergoing perinatal intimate examinations.

**Setting:**

Data were gathered in postnatal wards and Midwife Obstetric Units (MOUs) of level 1/district hospitals within the Tshwane District, Gauteng province, South Africa.

**Methods:**

Telephonic semi-structured interviews were conducted with 30 postnatal mothers from two district hospitals and four MOUs located in community health centres in the Tshwane District.

**Results:**

Women expressed the need for competent, empathetic health care providers who create a respectful, safe, and dignified environment. Minimising the frequency of intimate examinations reduced both physical and emotional discomfort. The use of alternative, less invasive assessment techniques enhanced women’s comfort and alleviated anxiety. Emotional and practical support from health professionals and significant others also improved women’s overall experience, decreasing fear and embarrassment.

**Conclusion:**

Intimate examinations often evoke fear and embarrassment due to privacy concerns and past trauma. Women require psychosocial support during these procedures. Midwives should receive training to conduct examinations with sensitivity and care to ensure women’s comfort and emotional safety.

**Contribution:**

The study provides South Africa-specific guidelines promoting holistic, respectful perinatal intimate examinations that emphasise communication, consent, and women’s psychosocial well-being.

## Introduction

The perinatal period is a time of increased vulnerability for women as they often experience stress, anxiety and psychological distress, particularly during intimate examinations, which may feel invasive and retraumatising for survivors of sexual or physical abuse (Zafra-Tanaka et al. 2019). The overuse of vaginal examinations beyond the World Health Organization’s (WHO’s) recommendation of every 6 h in the latent phase and 4 h from 5 cm to 8 cm followed by 2 h from 8 cm to 10 cm has been linked to increased maternal infections and discomfort (WHO 2018). Global guidelines advocate for trauma-informed, culturally sensitive and holistic care to address women’s psychological, social and physical needs (Avery et al. 2020; Clarke et al. 2021). However, many low-resource settings report abuse, neglect and the lack of respectful maternity care, which negatively impacts care-seeking behaviour and outcomes. Providing woman-centred, empathetic and holistic care supported by trained health care professionals is essential for improving maternal and neonatal outcomes (Dabson et al. 2021).

Intimate examinations are routine procedures performed by midwives, including breast, abdominal and vaginal examinations to exclude abnormalities (Bedaso et al. 2021). Some women may find it difficult to cooperate because of the sensitive nature of the examinations and potential embarrassment, anxiety and discomfort related to sexuality and the gender of novice practitioners (Bonnén et al. 2023). Breast examinations are associated with negative cues where some women believe that the breast is an intimate, personal organ and should not be exposed or touched (Davies, Lund & Schneider 2022). Other reasons can include religious and cultural differences, where women refuse to be examined by males and prefer female health care professionals (Janighorban, Kazemi & Haghani 2025).

Royal College of Obstetricians and Gynaecologists (2020) state that it depends on the preparedness and care that the patient is receiving during an examination; all practical measures to reduce the extent and duration of nudity should be considered, which do not jeopardise the thoroughness of the examination. The same applies to abdominal examinations as some women may be particularly sensitive about revealing various aspects of the body to a stranger, even a midwife, leading to a refusal of an examination or making it difficult to perform a detailed examination (Kumar, Saadaoui & Al Khodor 2022). This is also relevant to vaginal examination, which is described as intimate and intrusive in nature and associated with pain and discomfort (Zafra-Tanaka et al. 2019).

Psychosocial factors such as previous traumatic pregnancy experiences, lack of support, abandonment and a history of sexual or physical abuse are often overlooked in maternity care planning (Olza, Uvnäs-Moberg & Ekström-Bergström 2020). Women with a history of sexual abuse may find vaginal examinations distressing (Mayra et al. 2022). Building trust is essential for midwives and obstetricians to identify psychosocial issues affecting intimate examinations (Martinez-Vázquez et al. 2021). Women should be given safe opportunities to disclose underlying trauma or marital issues (Zafra-Tanaka et al. 2019), but many refrain because of unwelcoming and unsupportive clinical environments (Mkonyi et al. 2021). Such environments can retraumatise women with past abuse or negative maternity care experiences (Avanigadda & Kulasekaran 2021).

Mistreatment, such as slapping or hitting patients and the lack of respect during intimate examinations, makes patients feel vulnerable, dehumanised and worthless (Adu-Bonsaffoh et al. 2021) and prompts women to have other babies birthed outside the hospital (Mukanga et al. 2021). Midwives’ attitudes have a considerable influence on women’s behaviour during pregnancy and labour, which often leads to delays in appropriate and adequate care and which in turn increases risks of morbidity and mortality (Murugesu et al. 2021). Midwives need training to provide respectful maternity care, change their attitude and strengthen their professional ethics (Malatji & Madiba 2020). Women want their midwives to counsel them and focus more explicitly on their needs and interests (Edmaier & Pehlke-Milde 2024). They expect women monitoring during the perinatal period, which allows for a holistic stance and sensitivity for the individual. Midwives should provide holistic perinatal care by considering the connection between mind; body; emotions; social, cultural and environmental relationships; and past relationships to restore the woman as a whole (Minckas et al. 2021). Active communication between health care professionals and women is important to respond to women’s needs (Nerum et al. 2021), especially related to a sensitive topic such as intimate examinations during the perinatal period.

## Purpose of the study

This study seeks to explore the psychosocial needs of women undergoing perinatal intimate examinations.

### Theoretical framework

**FIGURE 1:**
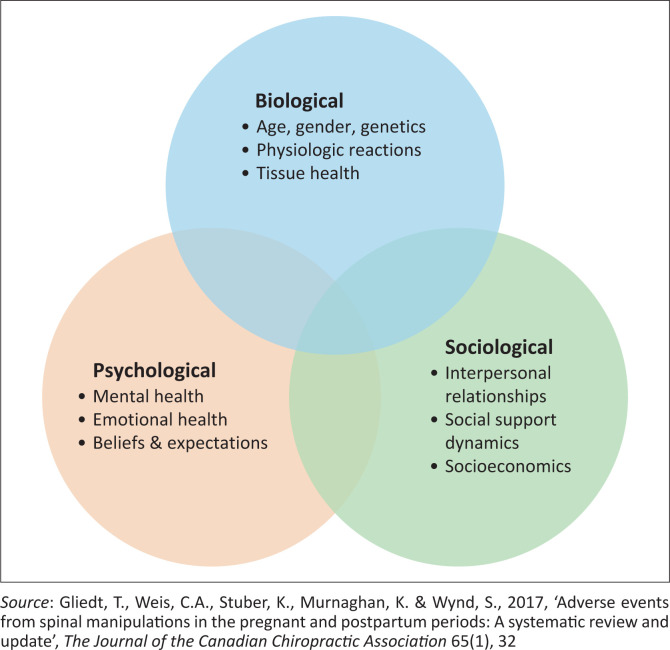
An illustration of biopsychosocial model comprising biological, psychological and sociological factors.

This study was informed by the biopsychosocial model developed by George L. Engel, which indicates that the cause, manifestation and outcome of wellness are determined by interactions between biological, psychological and social factors (Pereboom et al. 2023). Bolton and Gillert (2019) recommend that health care professionals should be cognisant of these three factors as they align with the rationale of multi-disciplinary teams and increasing recognition of the value of women’s views in providing good and effective health care. An inductive approach was used to describe the psychosocial needs of women undergoing intimate examinations (Ravi et al. 2024). The purpose of this model is to make midwives and health care professionals understand that in a pregnant woman, not only the biological factors but also the psychological and social factors should be considered, as they can influence the execution of intimate examinations (Shabot 2021) (see Figure 1).

## Research methods and design

### Research design and paradigm

The study was qualitative, exploratory and contextual in nature. An exploratory design was used to explore the psychosocial needs of women who underwent perinatal intimate examinations. The researcher explored a phenomenon using qualitative research methodology, as it is an approach for exploring and understanding the meaning that individuals or groups assign to a social or human problem. Furthermore, the study aimed to explore the phenomena from the viewpoint of the participants as they have experienced intimate examinations during their perinatal period. The exploratory nature of this study facilitated the researchers’ attainment of comprehensive insights and a profound understanding of the psychosocial needs of women. The design utilised the biopsychosocial model, which was proposed by George L. Engel, to explore the psychosocial needs of women during perinatal intimate examinations. The model indicates that the cause, manifestation and outcome of wellness are determined by interactions between biological, psychological and social factors (Pereboom et al. 2023). The purpose of this model is to make midwives and health care professionals understand that in a pregnant woman, not only the biological factors but also the psychological and social factors should be considered, as they can influence the execution of intimate examinations.

### Recruitment of participants

The researcher engaged potential participants directly in person to invite their voluntary involvement in the study. The researcher also asked the midwives to assist with the recruitment by inviting women who met the inclusion criteria to participate in the study before they were discharged. An information leaflet and consent form were provided to explain the purpose of the study, and permission was requested to obtain a telephone number so the researcher could contact them in the following week. The participants were asked to sign the information leaflet and consent form, and they received a written copy containing details of the study, as well as the researcher’s contact information. Recruitment and data collection were initiated after permission was obtained from the facilities’ management and the Research Ethics Committee of the Faculty of Health Sciences, University of Pretoria.

During recruitment, the participants had an opportunity to indicate their preference for a standard phone call, WhatsApp call, WhatsApp video call or another format, such as Google Meet and Zoom. Permission was obtained from the participants to audio record the telephonic interviews during the initial recruitment when they signed informed consent and were confirmed again at the beginning of the call.

### Population, sampling, inclusion and exclusion criteria

To obtain relevant and meaningful information that would generate rich data, effectively address the study’s objective and enhance trustworthiness, the researcher employed purposive sampling to recruit 30 postnatal women who had delivered vaginally at the time of the study in the selected district hospitals and Midwife Obstetric Units (MOUs) in a selected community health care centre. These participants took part in individual semi-structured telephonic interviews. The inclusion criteria were postnatal patients aged 18 years or older, who had given birth vaginally at the selected district hospitals and MOUs in the Tshwane District, Gauteng province, had no obstetric complications or emergencies and were willing to participate in the study. All women who did not meet the inclusion criteria were excluded from the study.

### Study setting

Data were collected in postnatal wards and MOUs of level 1/district hospitals in the Tshwane District in Gauteng province. Tshwane District is the metropolitan municipality that forms the local government of the northern Gauteng province in South Africa and is centred in the city of Pretoria, with surrounding towns and localities included in the local government area. The catchment population is 2 921 488. Tshwane District has four district hospitals, namely, Tshwane District Hospital, Pretoria West Hospital, ODI District Hospital and Jubilee District Hospital, as well as five MOUs situated in Eersterus, Dagga, Laudium, Soshanguve and Phedisong 4. The maternity wards in district hospitals provide 24 h services to low-risk pregnant women in labour and post-delivery care and are managed by obstetricians, midwives and midwife specialists (National Department of Health [NDOH] 2024). The selected district hospitals have, on average, a 24-bed capacity and manage 10 vaginal deliveries per day. The maternity staff work in shifts to have on average two medical officers, one specialist obstetrician, two midwife specialists and three midwives available at a time in a district hospital. All perinatal complications and emergencies are transferred to a tertiary hospital for further management.

### Data-collection tools and procedures

In this study, data were collected through a semi-structured telephonic interview guide, which included central and probing questions to direct the interviews. The interviews were conducted in English or in Northern Sotho, Southern Sotho, Zulu or Xhosa – languages in which the researcher was fluent. Data collection commenced after permission had been obtained from the facilities’ management and the Research Ethics Committee of the Faculty of Health Sciences, University of Pretoria. The researcher contacted the participants during the daytime, after the so-called blues on day 3, but before the memories of the intimate examinations became too vague – therefore, between day 4 and day 7 post-delivery. If a participant did not answer, the researcher attempted to call again after a few hours or the following day, but discontinued efforts after four unsuccessful attempts. Each telephonic interview was allocated approximately 30 min. Field notes were taken to keep track of the discussion. The interview guide was pilot-tested at a district hospital and the MOU on two voluntary postnatal women. The data were not included in the main study.

### Data analysis

In this study, data were analysed according to a method described by Busetto, Wick and Gumbinger (2020). The researcher listened to each audio tape and transcribed the data verbatim and labelled the field notes. Then, she read through all the data to obtain a general sense of information and reflect on its overall meaning, wrote notes in the margin as well as general thoughts about the data, read several participants’ transcripts and made a list of all topics that came to mind. The topics were typed into columns and labelled as codes, which were grouped into categories based on similarities and differences. Themes were developed from the categories to create headings in the report, and quotations were used to supplement the sub-themes. Six themes and 24 sub-themes emerged. This was followed up with a literature review to confirm or disconfirm the findings.

### Ethical considerations

An application for full ethical approval was made to the University of Pretoria, Faculty of Health Sciences Research Ethics Committee, and ethics consent was received on 11 April 2024. The ethics approval number is 149/2022, and it was obtained from the facility managers of the MOU and the Chief Executive Officers (CEOs) of the district hospitals in the selected District of Gauteng province, before data collection. In this study, the following will be applied:

Participants were protected through several ethical principles. They were assured of their right to protection from exploitation, meaning their participation would not expose them to harm, and any information they shared would be used solely for the study and never against them. The right to self-determination was upheld by obtaining voluntary informed consent, with participants made aware that they could withdraw at any time without consequences. The right to full disclosure was also respected, and participants were thoroughly informed about the study’s purpose, risks and benefits. They were not deceived, and the researcher explained that participation might evoke emotional discomfort, allowing sufficient time for questions and decision-making.

Furthermore, the right to fair treatment was observed by treating all participants, including those who declined or withdrew, with equal respect and without penalty. Participants were selected based on clear inclusion criteria, and no one was forced or required to justify their decision to opt out. Privacy was maintained by avoiding intrusive questions that could cause embarrassment or loss of dignity. Confidentiality and anonymity were ensured by not revealing participant identities at any stage of the research. Voice recordings and interview materials were securely stored, accessible only to the researcher and her supervisors, and personal data were kept separate from interview content using coded identifiers.

## Results

The findings, including identified themes and sub-themes, were systematically summarised and presented in Table 2. One primary theme, along with four sub-themes, emerged from the analysis. To ensure agreement, the coding and analytical processes were thoroughly reviewed and discussed multiple times. Direct quotes from participants were incorporated to reinforce the validity and transparency of the thematic selections. An integration of the literature review with the study findings was undertaken to deepen the interpretation and enhance the richness of the results. The data interpretation adhered to the methodology outlined by Busetto et al. (2020). A detailed discussion of the overall significance of the findings will be provided.

### Socio-demographic characteristics of participants

Thirty postnatal mothers who underwent vaginal delivery were interviewed through telephonic semi-structured interviews. Women were recruited in postnatal wards and MOUs of level 1/district hospitals in the Tshwane District in Gauteng province. All Participants resided in different regions of the Tshwane District. The participants’ ages ranged between 18 years and 38 years; two participants were below 20, 16 were between 20 years and 30 years old, and 12 were between 30 and 38 years old. The educational levels of the participants ranged from primary to tertiary; however, five participants had not attended school at all. Their occupational categories were grouped into two categories, government and private; eight of the participants were government workers, and 10 were self-employed. Six were working in the private sector, four were unemployed and two were scholars. Concerning marital status: 12 were married, 8 were cohabiting, 6 were separated from their partners and 4 were single in a relationship with their partners. Parity: 9 were primigravida and 21 were multigravida. Table 1 shows the demographic profile of the participants.

**TABLE 1:** Demographic profile of the participants.

Participant number	Age (years)	Gender	Parity	Employment status	Relationship status
P1	18	Female	G1P1	Scholar	Single
P2	34	Female	G2P2	Self-employed	Married
P3	37	Female	G2 P2	Self-employed	Separated
P4	33	Female	G1P1	Government worker	Married
P5	19	Female	G1P1	Self-employed	Single
P6	23	Female	G1P1	Private sector	Married
P7	20	Female	G1P1	Government worker	Single
P8	25	Female	G3P3	Private sector	Married
P9	29	Female	G1P1	Government worker	Separated
P10	22	Female	G1P1	Scholars	Single
P11	33	Female	G2P2	Government worker	Married
P12	38	Female	G3P3	Unemployed	Married
P13	22	Female	G1P1	Government worker	Cohabitating
P14	30	Female	G3P3	Government worker	Cohabitating
P15	27	Female	G2P2	Government worker	Cohabitating
P16	34	Female	G3P3	Self-employed	Cohabitating
P17	28	Female	G1P1	Private sector	Separated
P18	23	Female	G2P2	Self-employed	Cohabitating
P19	35	Female	G4P4	Private sector	Married
P20	24	Female	G2P2	Self-employed	Cohabitating
P21	34	Female	G3P3	Government worker	Married
P22	26	Female	G2P2	Self-employed	Separated
P23	28	Female	G3p3	Unemployed	Cohabitating
P24	36	Female	G2P2	Self-employed	Married
P25	29	Female	G3P3	Private sector	Separated
P26	24	Female	G3P3	Self-employed	Married
P27	27	Female	G2P2	Unemployed	Separated
P28	35	Female	G3P3	Self-employed	Married
P29	27	Female	G2P2	Unemployed	Cohabitating
P30	32	Female	G2P2	Private sector	Married

G, Gravida; P, Para.

The data were analysed, revealing one theme and four sub-themes, which are outlined in Table 2. These themes and sub-themes describe the psycho-social needs of women undergoing intimate examinations. The findings are further explored and supported by direct quotes in italics. To maintain anonymity, participants’ identities have been replaced with symbols as shown in brackets (e.g. P1). The findings are then followed by relevant discussions in literature.

**TABLE 2:** Psycho-social needs of women undergoing perinatal intimate examinations.

Themes	Sub-themes
1. Psychosocial needs of women undergoing intimate examinations	1.1. Sufficient and competent health care providers 1.2. Limited examinations 1.3. Alternative assessment methods 1.4. Support from others

### Theme 1: Psychosocial needs of women undergoing intimate examinations

This theme describes the psychosocial needs of women who underwent intimate examinations and has four sub-themes, namely, Sufficient and competent health care providers, Limited intimate examinations by health care providers, Alternative Assessment Methods, and Social Support from others.

#### Sub-theme 1.1: Sufficient and competent health care providers

The participants recommended that the Department of Health must recruit more midwives and obstetricians in maternity units as they are short-staffed and overwhelmed, which compromises the quality of health care that they are receiving and prevents them from receiving individualised care during intimate examinations. The participants indicate the following:

‘In that case, our government must hire more midwives and doctors to give us that quality care at times I understand that our hospitals are running short of midwives and they are overwhelmed and sometimes it is difficult for them to give all the patients equal attention but our care must not be compromised because of the staff shortage.’ (P1, 18 years old, Female, Scholar)

Another participant suggested that midwives should be given in-service training or counselling to be competent because they are going through a lot in maternity units, they can’t cope with the stress and workload:

‘Yes, or maybe they must be given refresher training or counselling because maybe they are going through a lot in the labour ward because even that day it was very busy for her, so I felt like she was taking out all her frustrations on us.’ (P16, 34 years old, Female, Government Worker)

Additionally, participants emphasised that midwives play a crucial role in protecting them from infections during intimate examinations. Regular and thorough handwashing is essential to prevent the transmission of harmful bacteria. One participant indicated the following:

‘Midwives should emphasise the importance of thorough handwashing before examinations to prevent any potential infections and be monitored to remove manicures during their shifts because they cause discomforts.’ (P27, 27 years old, Female, Unemployed)

#### Sub-theme 1.2: Limited examinations by health care providers

Limiting the number of vaginal examinations during labour is important to reduce physical discomfort, anxiety and to preserve patient dignity. Fewer examinations also lower the risk of infections, helping to create a safer and more supportive birthing environment. One participant stated the following:

‘I didn’t like to be checked more often, what is the reason for that … I believe when you are been checked once will be able to see how far I am and be able to predict when I am going to deliver … Instead of checking us many times. I was going to be happy if I was checked once not many times.’ (P16, 34 years old, Female, Government Worker)

‘Efforts should be made to reduce the frequency and invasiveness of examinations to alleviate unnecessary pain and discomfort and to limit the number of different healthcare professionals involved in examinations to reduce discomfort.’ (P4, 33 years old, Female, Government Worker)

A participant stated that she didn’t like to be examined by many health care providers:

‘I wished that I could have been examined by just one person. It’s difficult to be checked by many people who tell you different things. You kind of want to know where you are just to be in control of it and sort of to know how far you’ve potentially got. Thinking “Right, I’m nearly at the end.” Sort of an incentive to keep going.’ (P16, 34 years old, Female, Self-employed)

#### Sub-theme 1.3: Alternative assessment methods

Alternative assessment methods during intimate examinations can help ensure comfort, privacy and accuracy while minimising potential distress for women. The participants commented that they prefer foetal heart rate to be checked by an ultrasound scan because cardiotocograph (CTG) machines are uncomfortable. ultrasound scans, which often involve handheld probes, can be less invasive than CTG machines that require straps and monitoring belts, potentially allowing for a more comfortable experience. Using handheld devices can help maintain a sense of privacy and personal space during examinations, reducing feelings of exposure:

‘The machine that they are using to check babies is uncomfortable they must consider using other machines to check baby’s heart.’ (P8, 25 years old, Female, Private Sector)

Other participants stated that they also prefer another handheld device, such as a hand Doppler, which can help maintain a sense of privacy and personal space during examinations, reducing feelings of exposure. Another participant complained about the probes for the CTG machine that they are uncomfortable:

‘And they must try not to put the probes on the abdomen for a very long time or they should give us the option to choose whether we want to be checked by probes or other small machines.’ (P23, 28 years old, Female, Private Sector)

Others wished that there was an alternative method to check dilatation of the cervix other than the vaginal examination:

‘Yes, that examination is so intrusive and embarrassing, that I will never get used to it. This was my third delivery, and the experience was the same as the previous ones. I don’t know if midwives can use another method to check dilatation of the cervix.’ (P1, 18 years old, Female, Scholar)

#### Sub-theme 1.4: Support from others

Women often prefer to have emotional, psychological and physical support during intimate examinations. This support can come from a trusted friend, partner or health care professional as it helps create a more comfortable and reassuring environment. Having someone present can alleviate anxiety and make the experience feel less invasive, allowing women to feel more at ease and respected during the examination process. Two participants stated the following:

‘Allow more relatives to be present during labour and delivery, especially our mothers because they have experience and will be able to tell us what to do and not to do during labour.’ (P1, 18 years old, Female, Scholar)

‘At least if my mother was around, I would have felt better. They only allowed my husband to get into the room, not my mother. My husband knew nothing about delivery; he was as scared as me. I think maybe they should increase the number of relatives who want to be with you during delivery.’ (P3, 37 years old, Female, Self-employed)

Peer support plays a vital role during intimate examinations because peers who have undergone similar examinations can offer insights and share coping strategies. This shared experience can help normalise the situation, making it feel less isolating. The participant stated that their peers provided comfort and discussed the experience, which helped them to cope with the intimate examinations:

‘There was a lady in my room who was going to give birth as well but for her, it was for the second time. She told me that the pain was going to subside after the examination I had to relax my body during examinations and breathe in and out. She helped me a lot because I didn’t know what to do to ease the pain. [*Sigh*]’ (P18, 23 years old, Female, Self-employed)

The participants prefer to be supported by their mothers during intimate examinations more than by their partners; they wish their mothers could be given priority as chaperones:

‘I think it was going to be better if they allowed my mother to come in. They only allowed my husband to enter the room, not my Mom.’ (P20, 24 years old, Female, Self-employed)

The participants preferred their relatives to their partners:

‘Allow more relatives to be present during labour and delivery, especially our mothers because they have experience and will be able to tell us what to do and not to do during labour.’ (P28, 35 years old, Female, Self-employed)

Another reason that made participants request to have parents, relatives or significant others as their support system during labour is that midwives are always busy. The participants indicated the following:

‘No they are not small, my room was big to accommodate two people. Another reason is that midwives are always busy and doctors are not always available in the ward so most of the time I found myself being alone and frustrated.’ (P24, 36 years old, Female, Self-employed)

Another participant stated that she finds it difficult to tell the midwives about her social background or the abuse they experienced at home because they do not spend adequate time with them:

‘Sometimes it’s painful to tell them what you are experiencing in life because they are always in a hurry, and the more you are quite or not telling anyone about your situation is the more you going to feel stressed and more unwanted.’ (P21, 34 years old, Female, Self-employed)

‘I couldn’t tell the midwives about my history of sexual abuse because they were too serious focusing on my examinations which was also painful, it reminds me the day I was raped.’ (P5, 19 years old, Female, Self-employed)

## Discussion of the results

The study findings highlighted the psychosocial needs of women during perinatal intimate examinations. The participants consistently emphasised that their emotional, psychological and physical well-being was significantly influenced by how intimate care was provided. Many expressed the need for more compassionate, respectful and supportive practices. One of the dominant concerns raised by participants was the severe shortage of staff in maternity units. They recommended that the Department of Health recruit more midwives and obstetricians as current staffing levels were inadequate and overwhelmed personnel, which compromised the quality of health care services. These findings are supported by a study conducted by Kowalska et al. (2022), which confirmed that short-staffed maternity units often lead to poor quality of care. With insufficient staff, midwives may struggle to meet the needs of pregnant women and their newborns, resulting in longer waiting times and decreased monitoring during labour and delivery – factors that can ultimately compromise patient safety. Increasing the number of midwives and obstetricians could reduce complications, improve emotional support for mothers and enhance both maternal and neonatal care (Kowalska et al. 2022).

Furthermore, the findings showed that addressing staffing shortages is not only essential for safeguarding the health of mothers and babies but also for improving the overall efficiency and sustainability of the health care system. The participants believed that enhanced recruitment efforts, alongside the creation of supportive working environments, could significantly improve health outcomes and the satisfaction of both mothers and health care providers (Hofmeyr et al. 2019). Another recurring theme was the emotional strain experienced by midwives. One participant suggested that midwives should receive regular in-service training and counselling as they are often unable to cope with the high workload and emotional demands of maternity care. Counselling could offer midwives a safe space to share their feelings, helping to reduce burnout and emotional exhaustion (Hofmeyr et al. 2019). The participants noted that, in conjunction with training and mental health support, addressing staffing shortages would help ease midwives’ workload and ultimately enhance the quality of care provided during intimate examinations.

Infection prevention was also a key concern. The participants stressed that midwives play a crucial role in protecting them from infections during intimate procedures. They highlighted the importance of consistent and proper hand hygiene practices, such as washing hands with soap and water or using hand sanitiser before and after each examination. They also emphasised that midwives should maintain short, clean nails to minimise the risk of infections such as chorioamnionitis, which can arise from poor hygiene practices (Hofmeyr et al. 2019). Additionally, they reiterated the need for the Department of Health to urgently address the staffing challenges, noting that inadequate staffing directly impacts care quality and patient safety (De Klerk et al. 2019). Multiple studies have confirmed that improving staffing levels, enhancing training, providing emotional support and maintaining strict hygiene practices are all crucial measures that can significantly improve maternal and neonatal health outcomes (Adu-Bonsaffoh et al. 2021).

Several participants advocated for limiting the number of vaginal examinations during labour, emphasising the importance of preserving women’s comfort and dignity. This aligns with the WHO recommendations that stress the need to respect women’s preferences during childbirth (Gluck et al. 2020; WHO 2018). Excessive vaginal examinations have been associated with increased risks of infections, including chorioamnionitis and endometritis. Consequently, guidelines – such as South Africa’s recommendation of conducting examinations every 6 h during the latent phase (1 cm – 5 cm), every 4 h from 5 cm to 8 cm and every 2 h from 8 cm to 10 cm – have been introduced to mitigate these risks (NDOH 2024). The participants also expressed a preference for non-invasive methods to monitor labour, such as handheld ultrasound scans and Dopplers, which they felt preserved privacy and comfort better than traditional CTG machines. Research supports these preferences, showing that ultrasound and external observations, including physical indicators like the purple line, can be effective in tracking labour progression while respecting women’s comfort and right to informed choice (Avery et al. 2020; Pan et al. 2022; Papoutsis, Antonakou & Kourakos 2024).

Participants emphasised the need for emotional support during intimate examinations, preferring the presence of trusted companions, especially mothers, for comfort and reassurance, which helped reduce anxiety and made the experience feel more respectful (De Klerk et al. 2019). Peer support was also valued for providing coping strategies through shared experiences. However, many participants noted a lack of emotional support from midwives, often because of workload pressures. First-time mothers particularly desired clear communication and empathetic care, echoing literature that highlights the importance of emotional availability and empathy in improving patient satisfaction and outcomes (Bremnes et al. 2022).

## Trustworthiness of the study

The study ensured trustworthiness using Lincoln and Guba’s (1985) criteria: authenticity, credibility, dependability, confirmability and transferability. *Authenticity* was achieved by presenting participants’ psychosocial needs respectfully and making raw data available. *Credibility* was enhanced through prolonged engagement to understand the context deeply. *Dependability* was ensured by documenting processes thoroughly and maintaining regular supervision and safe storage of data. *Confirmability* was upheld by avoiding researcher bias, using an audit trail and accurately transcribing interviews. *Transferability* was supported through detailed contextual descriptions and a diverse sample of postnatal women.

### Limitation of the study

Conducting the study in the hospitals and midwives’ obstetric units in one District of Gauteng province was a limitation, as maternal and neonatal care is provided in the hospitals and midwives’ obstetric units in other districts in Gauteng province. As such, it would be beneficial to conduct this study in more than one district, as many women will be able to be reached. Furthermore, the implementation of these guidelines should be taken into consideration.

### Recommendations

To address the psychosocial needs of women during perinatal intimate examinations, it is recommended that midwives receive ongoing training in respectful communication, emotional support and consent-based care to enhance women’s psychological well-being. The Department of Health should urgently address staff shortages as adequate staffing allows midwives to provide more compassionate, patient-centred care during these sensitive procedures. Emotional support systems, such as allowing trusted companions or peer supporters to be present, should be integrated into routine care to reduce anxiety and promote dignity. Additionally, midwives must practice consistent infection prevention and minimise unnecessary vaginal examinations to protect both the physical and emotional safety of women during labour.

## Conclusion

In South Africa, many pregnant and postpartum women face significant psychosocial health challenges, often worsened by distressing experiences during perinatal intimate examinations. The study revealed that these examinations are frequently performed without adequate emotional support, contributing to anxiety, fear and potential postnatal depression. Addressing these psychosocial needs requires more trained staff, respectful care practices and emotional support for both patients and midwives. A holistic approach that considers both the physical and psychosocial well-being of women can greatly improve the quality and impact of maternal health care. To ensure a truly holistic perinatal care that includes emotional and psychological well-being, guidelines for respectful, consent-based perinatal examinations need to be developed (Gliedt et al. 2017) in the South African context.
